# Supplementary material for: Predicting visual function by interpreting a neuronal wiring diagram
Source: Nature. 2024 Oct 2;634(8032):113–23. doi: 10.1038/s41586-024-07953-5 (PMC11446822; doi:10.1038/s41586-024-07953-5)
Supplement: Supplementary file 6 — CRF and ERF predictions for individual TmY4 and TmY9 cells. Analogous to Supplementary Data 3, but for TmY target types. Shown are the top four monosynaptic pathways, the strongest pathway passing through each of the top ten intermediary types (ranking from Extended Data Fig. 7), and the trisynaptic pathway Tm1–TmY–Dm3–TmY (see the section entitled Prediction of spatial normalization). [file 41586_2024_7953_MOESM6_ESM.zip › DataS4/TmY9qΓèÑ/720575940630194171.pdf]

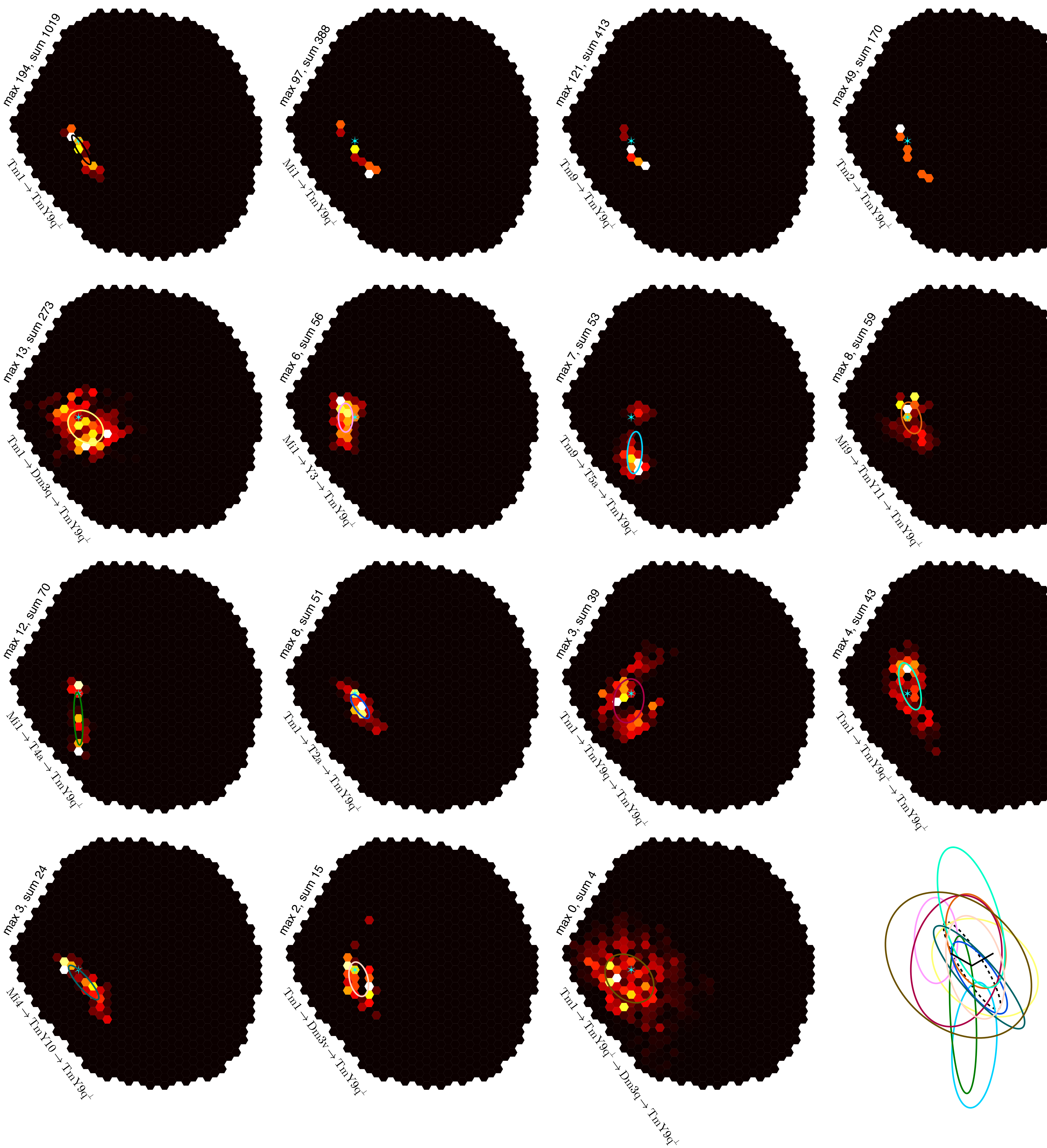

max 194, sum 1019
Tm1 → TmY9q⊥
max 97, sum 388
Mi1 → TmY9q⊥
max 121, sum 413
Tm9 → TmY9q⊥
max 49, sum 170
Tm2 → TmY9q⊥
max 13, sum 273
Tm1 → Dm3q → TmY9q⊥
max 6, sum 56
Mi1 → Y3 → TmY9q⊥
max 7, sum 53
Tm9 → T5a → TmY9q⊥
max 8, sum 59
Mi9 → TmY11 → TmY9q⊥
max 12, sum 70
Mi1 → T4a → TmY9q⊥
max 8, sum 51
Tm1 → T2a → TmY9q⊥
max 3, sum 39
Tm1 → TmY9q → TmY9q⊥
max 4, sum 43
Tm1 → TmY9q⊥ → TmY9q⊥
max 3, sum 24
Mi4 → TmY10 → TmY9q⊥
max 2, sum 15
Tm1 → Dm3v → TmY9q⊥
max 0, sum 4
Tm1 → TmY9q⊥ → Dm3q → TmY9q⊥
